# Singapore Declaration on Equitable Access to Health Information in the Western Pacific Region

**DOI:** 10.3352/jeehp.2009.6.7

**Published:** 2009-12-20

**Authors:** Asia Pacific Association of Medical Journal Editors

Following declaration was recommended to be published by member journals by Asia Pacific Association of Medical Journal Editors. Editorial office decided to publish it to propagate the spirit of this special declaration [1].

## CONSIDERING

That quality scientific and technical health information is essential for health policy makers, healthcare providers and health researchers to develop, improve, and implement efficient and effective healthcare systems and services.

That inequitable access to quality health information could result in poor health planning and healthcare delivery which adversely affect the health conditions of the public.

That surmounting this inequity requires public-private partnerships to facilitate equitable access to both production and consumption of health information for all.

That the Western Pacific Region Index Medicus (WPRIM), the Global Health Library (GHL), and the Asia Pacific Association of Medical Journal Editors (APAME) are important collaborative initiatives which are vital instruments to ensure the global accessibility and dissemination of quality health information in the Western Pacific Region.

## CONFIRM

Our commitment to free and universal dissemination and access to quality health information through the WPRIM and the GHL.

Our commitment to pursue the goals and objectives of APAME by further building networks, convening conferences, and organizing events to educate and empower editors, peer reviewers and authors in generating quality scientific and technical publications.

## CALL ON

Member States of the Western Pacific Region, in collaboration with stakeholders from the private sector, to formulate and implement policies that endorse free and equitable access to quality health information.

Stakeholders from the public and private sectors, national and international organizations, to support WPRIM and the GHL in order to ensure the free and global accessibility of health research done in the Western Pacific Region.

Governments, the private sector and other editors' associations to support APAME in implementing various activities, guidelines and practices that would improve the quality of scientific writing and publications n the Asia Pacific Region.

## COMMIT

Ourselves to persevere in the pursuit of the WPRIM and GHL initiatives through APAME, by encouraging peer-topeer relationships that will allow editors, editorial staff and librarians to maintain balance, work out ideas and provide mutual support.

Our organization, APAME, to building further networks, convening conferences, and organizing events to educate and empower editors, peer reviewers and authors to achieve and maintain internationally acceptable, but regionally realistic, scholarly standards.

This declaration was launched at the International Forum on Academic Medical Publishing held in conjunction with the Singapore Medical Journal Golden Jubilee Conference on November 6, 2009)

© Asia Pacific Association of Medical Journal Editors
